# Differential roles for cortical versus sub-cortical noradrenaline and modulation of impulsivity in the rat

**DOI:** 10.1007/s00213-016-4458-8

**Published:** 2016-10-15

**Authors:** Abigail Benn, Emma S. J. Robinson

**Affiliations:** School of Physiology, Pharmacology and Neuroscience, University of Bristol, Biomedical Sciences Building, University Walk, Bristol, BS8 1TD UK

**Keywords:** Atomoxetine, Amphetamine, Noradrenaline, Prefrontal cortex, Nucleus accumbens, Impulse control

## Abstract

**Rationale:**

Atomoxetine is a noradrenaline re-uptake inhibitor licensed for the treatment of adult and childhood attention deficit hyperactivity disorder. Although atomoxetine has established efficacy, the mechanisms which mediate its effects are not well understood.

**Objectives:**

In this study, we investigated the role of cortical versus sub-cortical noradrenaline by using focal dopamine beta hydroxylase-saporin-induced lesions, to the prefrontal cortex (*n* = 16) or nucleus accumbens shell (*n* = 18).

**Methods:**

Healthy animals were tested by using the forced-choice serial reaction time task to assess the impact of the lesion on baseline performance and the response to atomoxetine and the psychostimulant amphetamine.

**Results:**

We observed attenuation in the efficacy of atomoxetine in animals with lesions to the nucleus accumbens shell, but not the prefrontal cortex. Amphetamine-induced increases in premature responses were potentiated in animals with lesions to the prefrontal cortex, but not the nucleus accumbens shell.

**Conclusions:**

These data suggest that noradrenaline in the nucleus accumbens shell plays an important role in the effects of atomoxetine. Under these conditions, prefrontal cortex noradrenaline did not appear to contribute to atomoxetine’s effects suggesting a lack of cortical-mediated “top-down” modulation. Noradrenaline in the prefrontal cortex appears to contribute to the modulation of impulsive responding in amphetamine-treated animals, with a loss of noradrenaline associated with potentiation of its effects. These data demonstrate a potential dissociation between cortical and sub-cortical noradrenergic mechanisms and impulse control in terms of the actions of atomoxetine and amphetamine.

**Electronic supplementary material:**

The online version of this article (doi:10.1007/s00213-016-4458-8) contains supplementary material, which is available to authorized users.

## Introduction

Atomoxetine is a noradrenaline re-uptake inhibitor licensed for the treatment of adult and childhood attention deficit hyperactivity disorder (Christman et al. 2004; Michelson et al. 2002). In both receptor binding studies and in vivo functional characterization, atomoxetine has been shown to have a relatively selective effect on the noradrenaline transporter (NAT) versus either the dopamine active transporter (DAT) or serotonin transporter (SERT) (Bymaster et al. 2002; Seneca et al. 2006; Somkuwar et al. 2013), although see Ding et al. (2014) in relation to SERT occupancy. Although selective for the NAT, neurochemical studies suggest that atomoxetine also has effects on cortical dopamine as a result of the relatively low expression of DAT in the prefrontal cortex (PFC) and the role of NAT in terminating the effects of dopamine in this region (Bymaster et al. 2002). As well as effects on the catecholamine transmitters, atomoxetine also has a reasonable affinity for the NMDA receptor and may interact with this receptor at therapeutic doses (Di Miceli and Gronier 2015; Ludolph et al. 2010). Atomoxetine has also been shown to increase levels of acetylcholine at doses as low as 0.3 mg/kg (Tzavara et al. 2006). While the pharmacology of atomoxetine and other ADHD treatments is well characterized, the mechanisms which underlie their behavioral effects and efficacy are less well understood.

The link between catecholamines and ADHD is primarily a result of the drug treatments with limited direct neurobiological or genetic evidence for catecholamine dysfunction (Sun et al. 2014). A more consistent finding is that patients with ADHD have reduced volume and activity in prefrontal cortical regions which may lead to an imbalance or reduction in “top-down” modulation resulting in impulsivity and inattention. Some studies have also suggested that psychostimulant medications improve performance in normal volunteers particularly when they are fatigued suggesting that the efficacy of these drugs may not involve a direct interaction with a pathological state (Linssen et al. 2014). Whether or not catecholamines play a direct role in ADHD symptomology, understanding how treatments such as atomoxetine and psychostimulants achieve their beneficial effects could help in the development of improved therapies.

There is detailed literature on the effects of ADHD medications in humans and at the cellular, circuit, and behavioral level in animal studies (Berridge et al. 2006; Bymaster et al. 2002; Di Miceli and Gronier 2015; Tucha et al. 2006). Current theories propose that the stimulant and non-stimulant medications act through catecholaminergic mechanisms to improve prefrontal cortical function and both attention and impulse control (Arnsten 2000; Arnsten and Li 2005; Berridge et al. 2006). Tasks such as the five-choice serial reaction time task (5-CSRTT) have provided a valuable rodent model where both visuospatial attention and impulse control can be measured in rodents. In the 5-CSRTT, atomoxetine has been consistently shown to reduce premature responses (Blondeau and Dellu-Hagedorn 2007; Fernando et al. 2012; Robinson 2012; Robinson et al. 2008). Although the PFC has been the main focus of research, a study by Economidou et al. (2012) showed that the systemic effects of atomoxetine could be replicated in animals where the drug was directly infused into the nucleus accumbens shell (NAcSh) but not the core or PFC. In order to investigate this idea further, we utilized the dopamine beta hydroxylase (DβH), saporin-conjugated neurotoxin to induce noradrenergic lesions in the PFC or NAcSh (Milstein et al. 2007; Wrenn et al. 1996). In order to improve the sensitivity of our assay to detect a reduction in impulsive behavior, we used a modification of the 5-CSRTT, the forced-choice SRTT (F-CSRTT) which utilizes only a single response aperture (Murphy et al. 2008). It is hypothesized that removing the spatial unpredictability of the light cue increases the likelihood of an impulsive response (Murphy et al. 2008). In our laboratory, we observe levels of premature responses between 5 and 10 % while this is increased to ∼20 % in the forced-choice task (Robinson 2012). Once animals were trained in the task and the lesion had been allowed to establish, they were challenged by using a series of doses of atomoxetine based on previous studies (Robinson 2012; Robinson et al. 2008). We also wanted to test the effects of the lesions on the animal’s response to amphetamine. This drug has been shown to increase dopamine release in the nucleus accumbens core resulting in increased premature responding in the 5-CSRTT (Cole and Robbins 1989), an effect which is modified in animals with core versus shell neurotoxic lesions (Murphy et al. 2008). It was also of interest to see what effect, if any, amphetamine would have in the animals with PFC NA lesions as this may lead to an imbalance in the way amphetamine affects the levels of DA and NA and subsequent activation of post-synaptic alpha2 adrenoceptors versus D_1_ receptors.

## Methods

### Subjects

Subjects were two cohorts of male Lister hooded rats (*n* = 16 and *n* = 18) weighing approximately 250 g at the start of training (Harlan, UK). Rats were housed in groups of four with standard environmental enrichment (bedding, cardboard tubes) under temperature-controlled conditions and 12:12-h reverse light-dark cycle (lights off at 0700 h). Rats were food restricted to approximately 90 % of their free feeding weight (∼18 g/day laboratory chow, Purina, UK), with water provided ad libitum. Procedures were conducted in accordance with the requirements of the UK Animals (Scientific Procedures) Act 1986 and approved by the University of Bristol Animal Welfare and Ethical Review Board. Behavioral testing was carried out between 8 a.m. and 5 p.m. during the animals’ active phase.

### Forced-choice serial reaction time task training

**Fig. 1:**
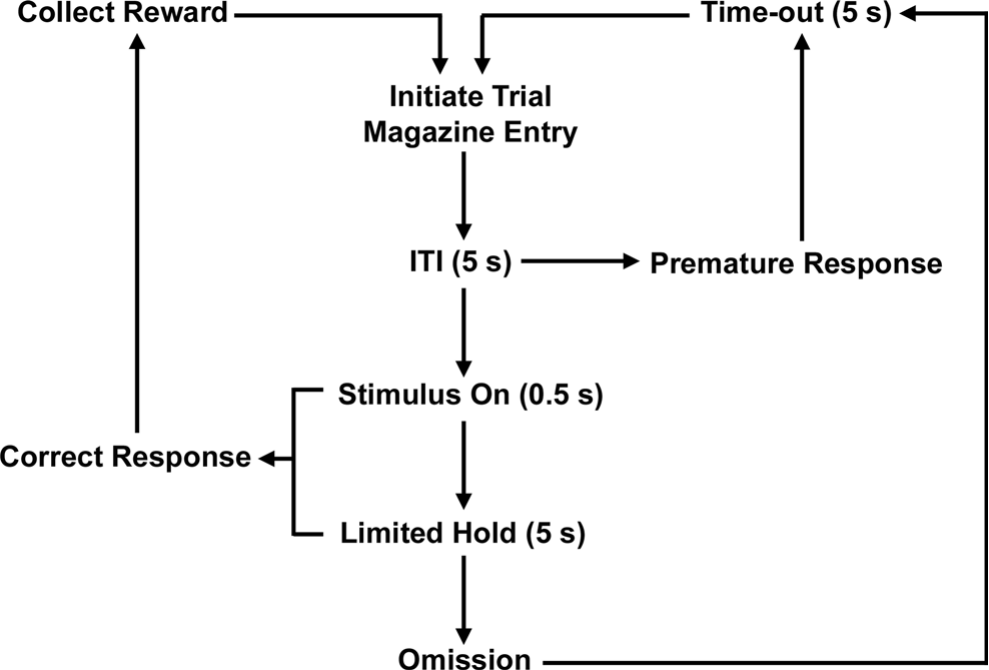
F-CSRTT trial sequence. Schematic illustrating the sequence of events for correct, omission, and premature trial types in the F-CSRTT, adapted from Bari et al. (2008)

Testing was carried out by using rat five-hole operant boxes (Med Associates, USA), controlled by KLimbic Software (Conclusive Solutions Ltd., UK). Subjects were trained to nose poke for a food reward (45 mg Noyes Precision Pellet, Sandown Scientific, UK) in response to a brief visual stimulus. Each trial was initiated by a nose poke in the magazine followed by a 5-s inter-trial interval (ITI). The light stimulus was then presented in the central aperture (0.5 s) followed a limited hold period (5 s). Only correct responses (response within the limited hold) were rewarded. In contrast to the 5-CSRTT, incorrect trials are not recorded in the F-CSRTT as only the central aperture is used, for task schematic see Fig. 1. Animals performed 100 trials per session (30-min duration), with the house light illuminated. Omissions (failure to respond within the limited hold period) and premature responses (response made during the ITI) were punished with a 5-s time-out and the house light switched off. Omissions are thought to be indicative of sensory, motor, or motivational factors as opposed to attentional accuracy (Robbins 2002). Premature responding provides a measure of response inhibition, whereas correct response latency (time from stimulus onset to a correct response) and magazine latency (time from a correct response to the reward collection) provide indices of motivational, sedative, or motor effects. This task differs to the 5-CSRTT in that animals are not required to attend to multiple locations as only one aperture is used (Dalley et al. 2004; Robbins 2002) causing more premature responses and greater sensitivity for detecting drug-induced reductions in impulsive responding (Murphy et al. 2008). Animals trained in the 5-CSRTT in our laboratory typically perform at a level of premature responses ∼5–10 % of the total trials performed (Benn and Robinson 2014; Robinson 2012), as opposed to ∼20 % for vehicle conditions in this study. Animals were trained according to a graduated protocol as described previously for the 5-CSRTT (Bari et al. 2008), until they had reached criterion (>80 correct trials, 0.5-s stimulus duration, 5-s ITI). No significant difference between three consecutive sessions was used to indicate stable baseline performance across task parameters (% correct, % omissions, % premature responses, correct and collection latencies). Baseline performance was analyzed preoperatively and postoperatively and before each dose response experiment.

### Surgical procedure

Following training, animals were assigned to surgical groups (lesion or vehicle) matched on baseline performance. Surgery was performed under aseptic conditions. Animals received bilateral lesions to the PFC (*n* = 16) or NAcSh (*n* = 18) by using stereotaxic injections of the immunotoxin anti-DβH-saporin (Advanced Targeting Systems, San Diego, USA) to selectively target noradrenergic afferent fibers (Milstein et al. 2007). Animals were anesthetized with inhaled isoflurane, placed in a stereotaxic frame (David Kopf Instruments, CA, USA), and fitted with a nose cone for continuous delivery of anesthetic. The skull was exposed and intraepicaine (2 %, Dechra Ltd., Staffordshire, UK) administered locally for postoperative analgesia. Two burr holes were drilled through the skull, and the immunotoxin or vehicle administered via a 22-gauge Hamilton syringe. The concentration of the immunotoxin for the PFC lesion was 0.04 μg/μL (dose = 0.02 μg in 0.5 μL per injection) (Milstein et al. 2007) and 0.02 μg/μL (dose = 0.004 μg in 0.2 μL per injection) for NAcSh lesions. Two injection sites per hemisphere were used to induce fiber loss across the extent of the PFC and shell sub-region. Sham-lesioned (sham) rats received vehicle injections of sterile PBS, pH 7.4. Stereotaxic coordinates used were (relative to bregma) as follows: PFC anteroposterior +2.7 and +3.4 mm, lateromedial ±0.6 mm, dorsoventral −3.6 mm and NAcSh anteroposterior +1.8 and +1.0 mm, lateromedial ±0.8 mm, dorsoventral −7.8 mm (Paxinos and Watson 2007). Following surgery, animals were housed in pairs and given 5–7-day recovery with free access to food and water. Animals were re-baselined for 10 sessions with stability confirmed by analysis of the last three baseline sessions (Supplementary Table S1).

### Task manipulations

Task manipulations (experiment 1), variable inter-trial interval (VITI) and noise distractor, were used to increase the demands of the task and assess whether the lesions had any effects on animals’ ability to respond to these different conditions. Previous studies by Milstein et al. (2007) used a similar approach and found effects for VITI but not for other conditions. Animals performed two consecutive days of baseline (5-s ITI) before each task manipulation. Each manipulation was used once to reduce habituation to the altered contingency. The VITI consisted of a 3, 4, 5, or 6-s ITI. Each ITI was represented equally throughout the session and presented randomly. The noise distractor consisted of a single burst of white noise (100 ms, 60 dB intensity) presented randomly during a 5-s ITI. Task manipulations were performed with the experimenter blind to the lesion status of the animal.

### Drugs

Atomoxetine hydrochloride (Tocris Bioscience, UK) and amphetamine (Sigma-Aldrich, UK) were dissolved in 0.9 % saline and administered by intraperitoneal injection in a final volume of 1 ml/kg; all drugs were prepared fresh each day. Drug doses used were based on previous studies by using similar behavioral tasks (Murphy et al. 2008; Robinson 2012; Robinson et al. 2008).

### Testing procedure

Animals received drug doses according to a fully randomized Latin-square design. Each drug day was preceded by a drug-free baseline test session, with each dose separated by a washout day (no testing or drugs). Atomoxetine dose response (experiment 2) was performed in the PFC group by using doses 0.3, 1.0, and 3.0 mg/kg (*t* = −40 min). As atomoxetine showed efficacy in this group at 0.3 mg/kg, the subsequent study in the NAcSh lesion group used a lower starting dose of 0.1, with 0.3, 1.0 mg/kg. For the amphetamine dose response (experiment 3), both cohorts received the same two doses (0.3, 1.0 mg/kg, i.p. *t = −*15 min). During amphetamine treatment, one animal from each cohort was removed from the final analysis due to a dosing error. Testing was performed with the experimenter blind to drug treatment and lesion status.

### DβH immunohistochemistry and image analysis

Lesion status was confirmed by DβH immunohistochemistry, following the completion of the behavioral experiments. Animals were anesthetized with sodium pentobarbitone and perfused with PBS followed by 4 % paraformaldehyde (0.1 M PB). The brains were removed, post-fixed in 4 % paraformaldehyde, and left to sink in 30 % sucrose solution. Brain sections (40 μm) were cut and stored in cryoprotectant (30 % sucrose, 30 % ethylene glycol, 0.1 M PB) at −20 °C until stained.

All brains were stained at the same time to minimize background variability across the groups. Incubations were performed at room temperature; each staining step was preceded by washes in 0.1 M PBS with 0.2 % Triton X-100 (PBS-T), unless otherwise stated. The staining procedure was as follows: 3 % hydrogen peroxide (10 min), blocking solution (3 % horse serum, 2 % bovine serum albumin, PBS-T, 30 min), DβH antibody solution (Merck Millipore, USA, MAB308, clone 4F10.2, 1:10,000, overnight), biotinylated horse anti-mouse IgG (Vector Labs, USA, 1:1000, 2–3 h), ExtrAvidin® solution (Sigma, UK, 1:1000, 2–3 h), and 3,3′-diaminobenzidine (DAB) solution (Vector Labs, USA, 1–2 min). Sections were mounted onto slides and cover slipped by using DPX. A negative control consisted of sections stained as above with the exclusion of the primary antibody.

Images were acquired by using a Leica DM IRBE inverted microscope attached to a cooled Hamamatsu CCD camera (Wolfson Bioimaging Facility, University of Bristol), with Volocity acquisition software (Improvision, PerkinElmer, USA). The area of DβH immunoreactivity, indicative of noradrenergic fibers, was measured by using ImageJ software (NIH) using the “Analyze Particles” function. TIFF images (1024 × 1024 pixels, 8 bits, ×20 magnification) were converted to binary counting masks after thresholding. Thresholding was based on the contrast in pixel intensity between specific staining and background and was consistent across brain regions. Threshold levels were checked by eye to ensure that fiber staining corresponded to the pixels selected by ImageJ (Lorentz et al. 2010; Matragrano et al. 2013). For each image, the area occupied by pixels with an intensity value above the background level was converted to mm^2^ and averaged across the group. Averaged values were used for brain regions where multiple stereotaxic levels had been analyzed (Supplementary Table S2). The experimenter was blind to the lesion status of the animal.

### Statistical analysis

All analyses were conducted by using SPSS for Windows (version 21, Chicago, USA). Sample size was based on previous studies by using similar tasks although the effect size for these novel experiments could not be reliably estimated. Given the subsequent results observed, the sample size for this type of interaction study may have been low and this should be considered for future experiments by using a similar approach. F-CSRTT performance measures recorded were % correct (correct responses divided by total number of correct and omissions), % omissions (omissions divided by total number of correct and omissions), % premature responses (premature responses divided by the total number of correct and omissions), correct latency (s), and collection latency (s). Dose response data for each drug were analyzed by using separate repeated-measures analysis of variance (RM-ANOVA), with dose as a within-subject factor and group (lesion or sham) as a between-subject factor. DβH immunostaining was analyzed by using RM-ANOVA with region as a within-subject factor and group as the between-subject factor. Reaction time data (correct latency) were tested for normality by using Kolmogorov-Smirnov test; no violations of normality were found (*p* > 0.05). Degrees of freedom were adjusted to more conservative values by using the Huynh-Feldt epsilon for instances of sphericity violation according to Mauchly’s test of sphericity. Epsilon values (*ɛ*) are stated where the degrees of freedom have been corrected. Significant main effects were further analyzed by post hoc comparisons between vehicle and drug doses by using least significant difference. The effect of task manipulations and preoperative/postoperative baseline performance were analyzed by using unpaired *t* tests between sham and lesion groups. Alpha level was set at 0.05, and graphs were plotted by using Prism 5.0 (GraphPad, USA).

## Results

### Quantification of DβH noradrenergic fibers

**Fig. 2:**
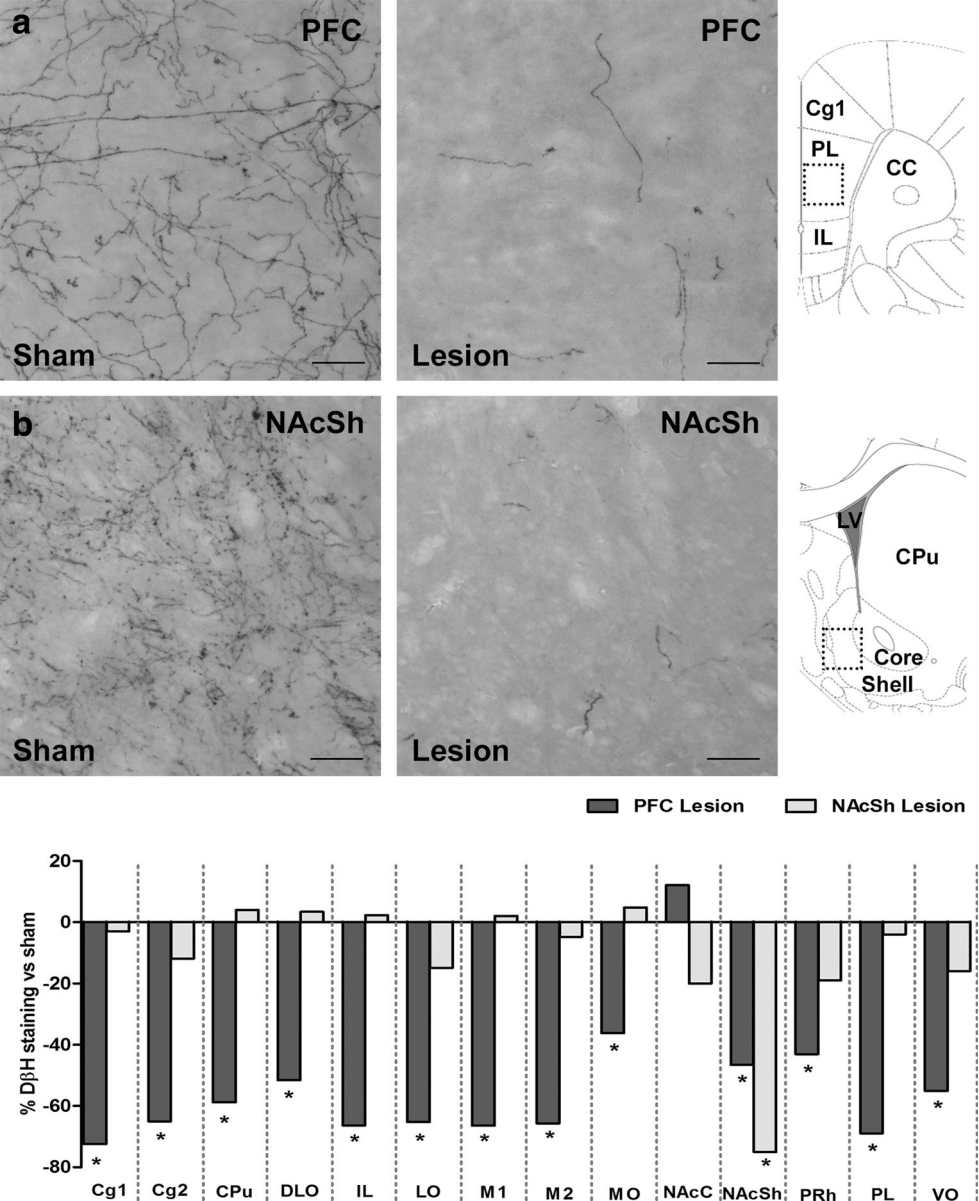
DβH immunostaining and lesion assessment. Representative images from the PFC (**a**) and NAcSh (**b**) showing reduced DβH fiber staining following DβH saporin lesions versus sham controls. Images shown are from the PL and NAcSh, *scale bar* = 50 μm; *black square* indicates approximate location on brain atlas. Lesion assessment summary (**c**) expressed as the percentage of DβH immunostaining compared to sham controls, see Table S2 for full list of brain regions analyzed. PFC (sham *n* = 8, lesion *n* = 8) and NAcSh (sham *n* = 9, lesion *n* = 9), *p < 0.05 sham versus lesion, within subject. *CC* corpus callosum, *Cg1* cingulate cortex 1, *Cg2* cingulate cortex 2, *CPu* caudate putamen, *DβH* dopamine beta hydroxylase, *DLO* dorsolateral orbital cortex, *IL* infralimbic, *LO* lateral orbital cortex, *LV* lateral ventricle, *M1* motor cortex, *M2* motor cortex, *MO* medial orbital cortex, *NAcC* nucleus accumbens core, *NAcSh* nucleus accumbens shell, *PL* prelimbic, *PRh* perirhinal cortex, *VO* ventral orbital cortex

DβH-saporin infusions in the PFC resulted in a noradrenergic lesion incorporating the cingulate, prelimbic, and infralimbic cortices (Fig. 2, Table S2, region × group *F*_(26, 364)_ = 6.55, *p* < 0.001, group *F*_(1, 14)_ = 25.50, *p* < 0.001). NA fiber loss extended into the adjacent orbital cortex, as well as the motor cortex and perirhinal cortex regions (Fig. 2, Table S2). A reduction in DβH immunoreactivity was also observed in more caudal regions, namely the caudate putamen and NAcSh (−60 % *p* = 0.003 and −47 % *p* = 0.002, respectively). Animals that had NAcSh DβH-saporin infusions were found to have selective lesions localized to the shell sub-region only (Fig. 2, Table S2; region × group *F*_(26, 416)_ = 3.67, *p* < 0.001, group *F*_(1, 16)_ = 0.05, *p* = 0.828).

### Pretesting baselines

No significant difference was observed across the last three consecutive baseline sessions pre or post surgery (Table S1). There were no differences between sham and lesion animals across performance variables for either PFC or NAcSh cohorts (Table S1) confirming that groups were matched prior to surgery, and lesions had no effect on baseline performance.

### Experiment 1: effects of task manipulations in animals with PFC or NAcSh noradrenergic lesions

Task performance under a VITI or noise distractor did not significantly differ between lesion and sham animals for either the PFC group (Table 1; VITI: all variables *t* < 1.47, *p* > 0.172, noise: all variables *t* < 1.49, *p* > 0.159) or NAcSh group (Table 1; VITI: all variables *t* < 0.92, *p* > 0.372, noise: all variables *t* < 1.25, *p* > 0.229, supplementary statistics Table S3).

**Table 1:** Task manipulations

Task manipulation	Group	Correct (%)	Omission (%)	Premature (%)	Correct latency (s)	Collection latency (s)
VITI	PFC sham	89.1 ± 1.6	10.9 ± 1.6	30.6 ± 4.8	1.58 ± 0.13	1.48 ± 0.11
	PFC lesion	90.8 ± 1.1	9.2 ± 1.1	22.7 ± 2.5	1.74 ± 0.07	1.36 ± 0.09
	NAcSh sham	92.6 ± 1.5	7.5 ± 1.5	45.9 ± 9.7	1.08 ± 0.11	1.58 ± 0.08
	NAcSh lesion	93.6 ± 1.5	6.4 ± 1.5	39.3 ± 3.9	1.07 ± 0.07	1.49 ± 0.06
Noise	PFC sham	88.6 ± 5.6	11.4 ± 5.6	25.2 ± 5.1	0.92 ± 0.08	1.56 ± 0.08
	PFC lesion	95.6 ± 0.6	4.4 ± 0.6	17.3 ± 1.6	1.07 ± 0.07	1.46 ± 0.10
	NAcSh sham	94.0 ± 1.4	6.0 ± 1.4	17.3 ± 3.3	0.94 ± 0.10	1.58 ± 0.08
	NAcSh lesion	93.4 ± 1.3	6.6 ± 1.3	24.3 ± 4.5	1.00 ± 0.07	1.49 ± 0.07

Performance in the F-CSRTT under VITI and noise distractor manipulations in animals with PFC or NAcSh noradrenergic lesions. Results are shown for the total population, mean ± SEM, PFC *n* = 16, NAcSh *n* = 18, animals per group

### Experiment 2: effects of atomoxetine treatment in PFC versus NAcSh lesion animals

#### PFC lesions

**Fig. 3:**
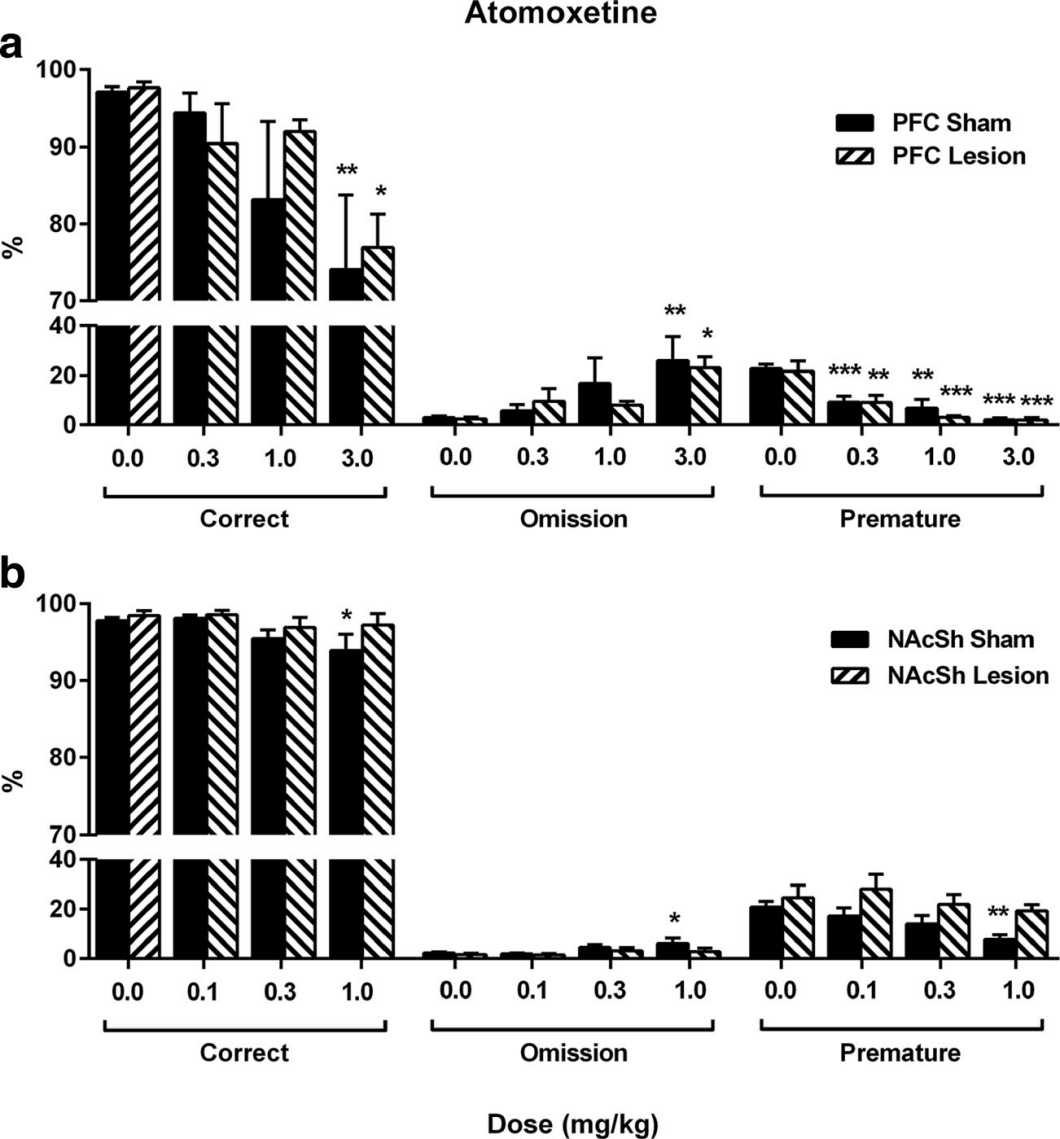
Atomoxetine dose response. The effects of atomoxetine (0.0–3.0 mg/kg) on F-CSRTT performance in animals with PFC (**a**) or NAcSh (**b**) noradrenergic lesions. Results are shown for the total population, mean ± SEM, *n* = 16 (PFC lesions) and *n* = 18 (NAcSh lesions) animals per group. *p < 0.05, **p < 0.01, ***p < 0.001, versus vehicle (within subject)

Atomoxetine treatment reduced premature responses at all doses tested for sham and lesion animals (Fig. 3a; *F*_(3.0, 42.0)_ = 29.65, *p* < 0.001). Correct responses were reduced (Fig. 3a; *F*_(2.2, 30.9)_ = 9.80, *p* < 0.001, *ɛ* = 0.74) with an increase in omissions (*F*_(2.2, 30.9)_ = 9.80, *p* < 0.001, *ɛ* = 0.74) at the highest dose tested in sham (3.0 mg/kg, *p* = 0.007) and lesion animals (3.0 mg/kg, *p* = 0.013). A significant effect of dose on correct latency and collection latency was also observed (Table 2; *F*_(3.0, 42.0)_ = 27.41, *p* < 0.001 and *F*_(3.0, 42.0)_ = 16.85, *p* < 0.001, respectively), with increased correct latency in sham animals (1.0 mg/kg *p* = 0.002, 3.0 mg/kg *p* < 0.001) and across all doses in lesion animals (0.3 mg/kg *p* = 0.027, 1.0 mg/kg *p* = 0.008, 3.0 mg/kg *p* < 0.001). Collection latency was also increased in sham (Table 2; 0.3 mg/kg *p* = 0.024, 1.0 mg/kg *p* = 0.003, 3.0 mg/kg *p* = 0.001) and lesion animals (1.0 mg/kg *p* = 0.029, 3.0 mg/kg *p* = 0.002). No group effect or dose × group interaction were found for any performance variables (Fig. 3a, Table 2; *F*_(1.0, 14.0)_ < 1.12, *p* > 0.307, and *F*_(3.0, 42.0)_ < 0.79, *p* > 0.454, supplementary statistics Table S4).

**Table 2:** F-CSRTT latency data

Treatment	Group	Dose (mg/kg)	Correct latency (s)	Collection latency (s)
Atomoxetine	PFC sham	0.0	0.83 ± 0.07	1.56 ± 0.10
		0.3	1.20 ± 0.24	1.71 ± 0.13*
		1.0	1.64 ± 0.27**	1.93 ± 0.19**
		3.0	2.34 ± 0.41***	2.00 ± 0.14**
	PFC lesion	0.0	0.92 ± 0.09	1.44 ± 0.06
		0.3	1.37 ± 0.24*	1.54 ± 0.09
		1.0	1.57 ± 0.15**	1.69 ± 0.13*
		3.0	2.43 ± 0.21***	1.82 ± 0.14**
	NAcSh sham	0.0	0.86 ± 0.09	1.57 ± 0.08
		0.1	0.95 ± 0.10	1.58 ± 0.07
		0.3	1.14 ± 0.12*	1.66 ± 0.10
		1.0	1.31 ± 0.14**	1.95 ± 0.14***
	NAcSh lesion	0.0	0.78 ± 0.09	1.47 ± 0.05
		0.1	0.75 ± 0.05	1.56 ± 0.06
		0.3	0.89 ± 0.08	1.53 ± 0.06
		1.0	1.01 ± 0.13	1.74 ± 0.08**
Amphetamine	PFC sham	0.0	0.91 ± 0.07	1.57 ± 0.10
		0.3	0.72 ± 0.10	1.50 ± 0.07
		1.0	1.25 ± 0.16*	1.58 ± 0.20
	PFC lesion	0.0	0.99 ± 0.11	1.43 ± 0.06
		0.3	0.82 ± 0.11	1.35 ± 0.05
		1.0	1.16 ± 0.10	1.51 ± 0.22
	NAcSh sham	0.0	0.93 ± 0.10	1.56 ± 0.06
		0.3	0.80 ± 0.12	1.45 ± 0.10
		1.0	0.89 ± 0.13	1.43 ± 0.10
	NAcSh lesion	0.0	0.89 ± 0.11	1.52 ± 0.08
		0.3	0.71 ± 0.05	1.33 ± 0.06**
		1.0	1.24 ± 0.17	1.37 ± 0.04*

The effects of atomoxetine (0.0–3.0 mg/kg) and amphetamine (0.0–1.0 mg/kg) on latency in animals with PFC or NAcSh noradrenergic lesions. Results are shown for the total population, mean ± SEM, PFC *n* = 16 (amphetamine *n* = 15), NAcSh *n* = 18 (amphetamine *n* = 17). *p < 0.05, **p < 0.01, ***p < 0.001, versus vehicle (within subject)

#### NAcSh lesions

Atomoxetine treatment caused a reduction in premature responses at the highest dose in sham (Fig. 3b; *F*_(2.6, 41.9)_ = 5.73, *p* = 0.003, *ɛ* = 0.87, 1.0 mg/kg *p* = 0.001) but not lesion animals (0.1 mg/kg *p* = 0.456, 0.3 mg/kg *p* = 0.557, 1.0 mg/kg *p* = 0.118). No group effect (*F*_(1.0, 16.0)_ = 3.90, *p* = 0.067) or dose × group interaction (*F*_(2.6, 41.9)_ = 0.93, *p* = 0.425, *ɛ* = 0.87) were found for premature responses. Correct responses were reduced, and omissions increased in sham animals at the highest dose (Fig. 3b; *F*_(2.2, 34.6)_ = 4.00, *p* = 0.025, *ɛ* = 0.72, 1.0 mg/kg *p* = 0.043); however, no effect was found in lesion animals (0.1 mg/kg *p* = 0.851, 0.3 mg/kg *p* = 0.214, 1.0 mg/kg *p* = 0.500). Atomoxetine treatment also increased correct latency in sham animals only (Table 2; correct latency *F*_(3.0, 48.0)_ = 10.30, *p* < 0.001, 0.3 mg/kg *p* = 0.016, 1.0 mg/kg *p* = 0.002) with collection latency increased across both groups at the highest dose (Table 2; *F*_(3.0, 48.0)_ = 13.41, *p* < 0.001, 1.0 mg/kg *p* < 0.001). No group effect or dose × group interaction were found for any performance variables (Fig. 3b, Table 2; *F* < 1.74, *p* > 0.104, and *F* < 2.98, *p* > 0.104, supplementary statistics Table S4).

### Experiment 3: effects of amphetamine treatment in PFC versus NAcSh lesion animals

One animal from the PFC and NAcSh groups was removed from the final analysis for amphetamine due to a dosing error.

#### PFC lesions

**Fig. 4:**
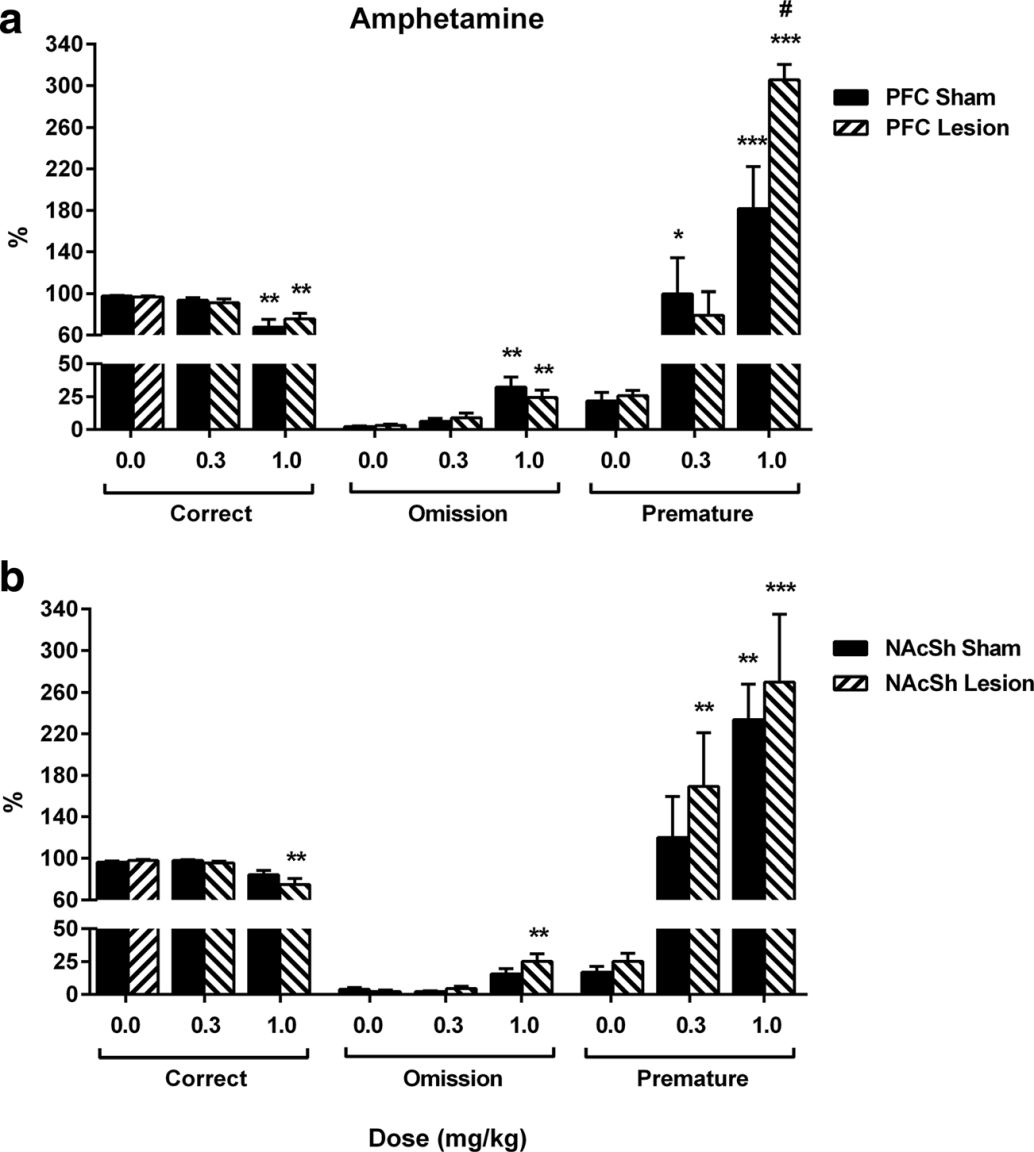
Amphetamine dose response. The effects of amphetamine (0.0–1.0 mg/kg) on F-CSRTT performance in animals with PFC (**a**) or NAcSh (**b**) noradrenergic lesions. Results are shown for the total population, mean ± SEM, *n* = 15 (PFC lesions) and *n* = 17 (NAcSh lesions) animals per group. *p < 0.05, **p < 0.01, ***p < 0.001, versus vehicle (within subject); #*p* < 0.05 versus sham (between subject)

Amphetamine treatment increased premature responding in a dose-dependent manner in sham animals (Fig. 4a; *F*_(2.0, 26.0)_ = 47.26, *p* < 0.001, 1.0 mg/kg *p* = 0.027, 0.3 mg/kg *p* < 0.001) and at the highest dose in lesion animals (0.3 mg/kg *p* < 0.001). The effect was potentiated in lesion animals compared to sham animals (Fig. 4a; 1.0 mg/kg *p* = 0.010) with a dose × group interaction (*F*_(2.0, 26.0)_ = 5.64, *p* = 0.009) but not a group effect found (*F*_(1.0, 13.0)_ = 3.14, *p* = 0.100). In sham and PFC lesion animals, amphetamine reduced correct responses at the highest dose (Fig. 4a; dose *F*_(1.5, 19.4)_ = 22.42, *p* < 0.001, *ε* = 0.75, 0.3 mg/kg *p* = 0.001) and increased omissions (dose *F*_(1.5, 19.4)_ = 22.42, *p* < 0.001, *ε* = 0.75, 0.3 mg/kg *p* = 0.001). Correct latency was also affected by amphetamine treatment (Table 2; dose *F*_(2.0, 26.0)_ = 14.87, *p* < 0.001), with an increase at the highest dose in sham animals (1.0 mg/kg *p* = 0.011) but no effects observed in lesion animals (0.3 mg/kg *p* = 0.109, 1.0 mg/kg *p* = 0.159). Collection latency was unaffected by amphetamine treatment (Table 2; dose *F*_(1.3, 17.4)_ = 0.51, *p* = 0.538, *ε* = 0.67). No group effect or dose × group interaction were found for any other performance variables (Fig. 4a; Table 2; *F* < 3.14, *p* > 0.10, and *F* < 0.95, *p* > 0.378, supplementary statistics Table S4).

#### NAcSh lesions

Amphetamine treatment affected premature responses (Fig. 4b; dose *F*_(1.6, 24.4)_ = 14.43, *p* < 0.001, *ε* = 0.81), with increases observed at 1.0 mg/kg in sham animals (*p* = 0.001, 0.3 mg/kg *p* = 0.062) and at all doses in lesion animals (0.3 mg/kg *p* = 0.006, 1.0 mg/kg *p* < 0.001). The highest dose also reduced correct responses (Fig. 4b; dose *F*_(1.3, 19.0)_ = 18.58, *p* < 0.001, *ε* = 0.63) and increased omissions (*F*_(1.3, 19.0)_ = 18.58, *p* < 0.001) although these effects were only significant for the lesion animals at 1.0 mg/kg (*p* = 0.001), but not sham animals (*p* = 0.079). Collection latency was reduced in lesion animals at both doses (Table 2; dose *F*_(2.0, 30.0)_ = 6.89, *p* = 0.003, 0.3 mg/kg *p* = 0.009, 1.0 mg/kg *p* = 0.021) but not sham animals (0.3 mg/kg *p* = 0.161, 1.0 mg/kg *p* = 0.121). A main effect of DOSE was also observed for correct latency (Table 2; *F*_(1.6, 24.7)_ = 4.85, *p* = 0.022); however, post hoc tests revealed no specific effects for sham or lesion animals at any doses (*p* > 0.106). No group effect or dose × group interaction were found for any performance variables (Fig. 4b, Table 2; *F* < 1.36, *p* > 0.221, and *F* < 3.23, *p* > 0.18, supplementary statistics Table S4).

## Discussion

These studies support the hypothesis that an important loci for the effects of atomoxetine on impulse control in the rat is the NAcSh and involves noradrenaline. Lesions to the NAcSh attenuated not only atomoxetine’s effects on premature responses but also its effects on other task variables suggesting that these may all also be linked to a noradrenergic mechanism. Although noradrenaline in the PFC did not appear to contribute to the efficacy of atomoxetine, the effects of amphetamine were potentiated in animals with lesions suggesting that PFC noradrenaline provides some degree of modulatory control under this condition. In removing the noradrenergic input to the PFC, we may have created an imbalance in noradrenergic versus dopaminergic modulation meaning that the effects of amphetamine were potentiated (Arnsten 2011). Unlike previous studies using the 5-CSRTT (Milstein et al. 2007), we did not observe any differences in animal performance under baseline conditions or when challenged by using a VITI or noise distractor. The following discussion considers the main findings and their implications for the role of noradrenaline in impulse control and atomoxetine’s efficacy.

Current theories relating to the role of catecholamines in ADHD have focused on the PFC and the proposal that noradrenaline, via alpha_2_ adrenoceptors, and dopamine, via D_1_ receptors, provide modulatory input which regulates neuronal firing (Arnsten and Dudley 2005). If there is an imbalance in the input from these transmitters, impairments in attention and impulse control, as well as working memory deficits, are observed (Arnsten et al. 1988, 1998; Harrison et al. 1997; Sawaguchi et al. 1988; Sirvio et al. 1994; Steere and Arnsten 1997). It is also proposed that psychostimulants, atomoxetine, and alpha_2_ adrenoceptor agonists such as guanfacine remediate the imbalance reducing the symptoms of ADHD (Arnsten and Dudley 2005; Bari et al. 2009; Berridge et al. 2006; Pattij et al. 2012). With PFC lesions, we failed to observe any difference in the actions of atomoxetine with both sham and lesion animals, exhibiting improved impulse control in a dose-dependent manner similar to previous studies (Fernando et al. 2012; Navarra et al. 2008; Robinson 2012). At the higher doses tested (1.0 and 3.0 mg/kg), we also observed more general effects on task performance with an increase in omissions and decrease in correct responses. Response latencies were also increased in both groups. These findings would suggest that atomoxetine does not act via cortical noradrenaline but, as discussed below, involves actions within sub-cortical structures. However, despite the widespread lesion which we achieved in the PFC, this is not a total lesion, and some noradrenaline fibers remain. The selectivity of the DβH-saporin immunotoxin has been confirmed previously, with no depletion of dopamine or monoaminergic neurons that do not express DβH (Milstein et al. 2007; Pickel et al. 1975; Wrenn et al. 1996). It is therefore likely that cortical dopamine, and any effects of atomoxetine mediated via this transmitter, would have been preserved. While our lesion of the NAcSh was very selective, the PFC lesion was more extensive and some fiber loss was also observed in the NAcSh. The level of loss was ∼47 %, while the NAcSh only lesions achieved a loss of ∼75 %. Given the behavioral data we observed, it would appear that some loss of noradrenaline from the NAcSh can occur without impact on atomoxetine’s effects but the 75 % loss observed in the NAcSh lesion rats is associated with an attenuation in efficacy. However, we cannot preclude the possibility that concurrent loss of NA from PFC and NAcSh prevented a PFC-mediated mechanism from being observed.

The results from the NAcSh lesion animals would suggest that noradrenaline in this region plays an important role in mediating atomoxetine’s effects on impulsivity. Previous studies support this as targeted infusions of atomoxetine into the shell but not the core or PFC have yielded similar findings (Economidou et al. 2012). Our work refines this further by showing that the effects are dependent on an intact noradrenergic system. The noradrenergic input to the nucleus accumbens region and basal ganglia as a whole is limited, and previous work has largely overlooked this region in terms of noradrenaline, much more commonly linking it to dopamine. The NAcSh appears to receive a relatively large noradrenergic input with our immunohistochemical analysis showing fibers as well as terminal fields and a density of DβH staining approximately two thirds of the level in the PFC. In contrast, the nucleus accumbens core and caudate nucleus have very low levels (Berridge et al. 1997). The source of noradrenaline to the NAcSh is still not fully understood, and few studies have investigated this, but there has been one paper at least which proposes that the noradrenaline neurons arise in the nucleus tractus solitaries rather than the locus coeruleus (Delfs et al. 1998). From our data, it appears that noradrenaline in the NAcSh is exerting some degree of modulatory influence although further studies are needed to elucidate the exact mechanism.

From our behavioral data, we observed both a reduction in atomoxetine’s effects on premature responding but also its more general effects on motivation, suggesting a possible link between motivation and impulsivity mediated by NAcSh noradrenaline. In general, the nucleus accumbens core and dopamine release within this region have been linked to impulsivity, particularly in the 5-CSRTT (Besson et al. 2010; Murphy et al. 2008). The NAcSh region has been less well characterized in models of impulsivity but has been studied extensively in motivation (Pecina et al. 2006; Reynolds and Berridge 2002). Bringing these ideas together with our behavioral data, we hypothesize that noradrenaline in the NAcSh is acting to regulate the motivational state and arousal which underlies the premature response in the F-CSRTT. If this is the case, then atomoxetine may facilitate this noradrenergic mechanism resulting in improved control in animals presented with a cue associated with responding for reward.

The effects of amphetamine were also interesting and revealed a dissociation between the different lesion groups. No effects were observed in NAcSh-lesioned animals; however, animals with a PFC noradrenaline lesion exhibited a potentiated response to amphetamine. A link between the nucleus accumbens core and shell and impulsive behavior in response to amphetamine has previously been shown by Murphy et al. (2008). In this study, NAcSh lesions were associated with an attenuated response to amphetamine, while lesions to the core resulted in a potentiated effect on premature responses (Murphy et al. 2008). However, our data suggest that removal of only noradrenergic inputs to the NAcSh region does not have any effects on performance in the F-CSRTT under baseline conditions or during challenging conditions (amphetamine, VITI, noise). In contrast, PFC noradrenaline appears to contribute in some way to modulating the effects of amphetamine, and our data would suggest that this is via an inhibitory role. The most likely explanation for this is the hypothesis that both NA and DA act together in the PFC to regulate behavior. A balance between alpha2 and dopamine D1 activation helps to optimize PFC neuronal function and cognition (Arnsten and Dudley 2005). By removing the noradrenergic input to the PFC and administering amphetamine, the rise in catecholamine transmission in the PFC would be limited to dopamine and result in excessive activation of D_1_ receptors. A large body of work has shown that the balance between DA and NA in the PFC plays a critical role in cognition and the actions of psychostimulants (Arnsten and Dudley 2005; Berridge and Arnsten 2012; Milstein et al. 2010; Schmeichel and Berridge 2013; Spencer et al. 2015).

The dissociation between the behavioral effects of amphetamine and atomoxetine in the different lesion groups may have relevance to the clinical situation, as responder efficacy has been linked to the type of ADHD (hyperactive/impulsive, inattentive, combined) as well as different functional outcomes (Fredriksen et al. 2013; Newcorn et al. 2008). Our results suggest that amphetamine and atomoxetine differentially interact with the NA system when different regions are impaired, something which may also be relevant to the clinical presentation of ADHD. Although, it should be noted that this work was done in normal rats by using a dose range consistent with the recommended daily dose prescribed in patients (Childress and Berry 2012; Sharma and Couture 2014). Further studies to investigate these findings and understand more about the underlying neurobiology may help in providing better management of patients with the most appropriate treatments. Although these data suggests dissociation between cortical (PFC) versus sub-cortical (NAcSh) noradrenaline in impulse control, this work does not preclude other actions of atomoxetine (e.g., increasing cortical acetylcholine) or provide insight into the attentional effects of the drug (Tzavara et al. 2006). Several limitations of the study also need to be taken into consideration. We cannot preclude the possibility that concurrent damage to sub-cortical structures in the PFC-lesioned animals may have precluded a specific effect of atomoxetine in these animals. However, our study assessed fiber loss across a wider range of brain regions by using a quantitative approach, which may have uncovered a greater extent of fiber loss than previously shown (Milstein et al. 2007).

Our baseline premature responses in the F-CSRTT are higher than those typically observed in our 5-CSRTT (Benn and Robinson 2014; Robinson 2012), although the levels observed in this study were much lower than those previously reported for the F-CSRTT (Murphy et al. 2008). Alternative methods to increase premature responding could be used in future studies such as a long inter-trial interval (Economidou et al. 2012). If baseline premature responding were higher, this might also increase the effect size and help address the issues seen in this study around power. It may also be necessary to increase the same size when looking at these interactions between lesion and drug effects. At the doses tested, no effects of atomoxetine were observed in NAcSh lesion animals although we tested a lower dose range in this group to try to maximize our opportunity for seeing an effect. If we tested higher doses, we may also see effects in the NAcSh lesion animals arising either from actions in other brain regions associated with the effects of the drug on the residual noradrenaline fibers. We also need to consider the impact of the PFC lesions on shell NA innervations.

## Electronic supplementary material

Table S1 (DOC 43 kb)

Table S2 (DOC 79 kb)

Table S3 (DOC 44 kb)

Table S4 (DOC 63 kb)
