# Supplementary material for: Differential roles for cortical versus sub-cortical noradrenaline and modulation of impulsivity in the rat
Source: Psychopharmacology (Berl). 2016 Oct 15;234(2):255–66. doi: 10.1007/s00213-016-4458-8 (PMC5203835; doi:10.1007/s00213-016-4458-8)
Supplement: Supplementary file 1 — (DOC 43 kb) [file 213_2016_4458_MOESM1_ESM.doc]

Table S1: Pre- and post-operative baseline data

|  | Correct (%) | | Omission (%) | | Premature (%) | | Correct Latency (s) | | Collection Latency (s) | |
| --- | --- | --- | --- | --- | --- | --- | --- | --- | --- | --- |
| Group | Pre- | Post- | Pre- | Post- | Pre- | Post- | Pre- | Post- | Pre- | Post- |
| **PFC** |  |  |  |  |  |  |  |  |  |  |
| Sham | 93.6 ± 1.5 | 94.7 ± 1.3 | 6.4 ± 1.5 | 5.3 ± 1.3 | 15.4 ± 3.2 | 21.3 ± 2.6 | 0.95 ± 0.25 | 0.78 ± 0.07 | 1.67 ± 0.10 | 1.50 ± 0.13 |
| Lesion | 94.3 ± 1.4 | 95.5 ± 1.0 | 5.7 ± 1.4 | 4.5 ± 1.0 | 15.0 ± 2.7 | 19.4 ± 2.4 | 0.82 ± 0.29 | 0.75 ± 0.06 | 1.42 ± 0.09 | 1.41 ± 0.11 |
| **NAcSh** |  |  |  |  |  |  |  |  |  |  |
| Sham | 91.4 ± 1.7 | 92.6 ± 1.2 | 8.7 ± 1.7 | 7.4 ± 1.2 | 17.6 ± 2.3 | 13.3 ± 2.7 | 1.09 ± 0.10 | 1.12 ± 0.12 | 1.73 ± 0.10 | 1.73 ± 0.09 |
| Lesion | 92.3 ± 1.8 | 93.9 ± 1.0 | 7.7 ± 1.8 | 6.1 ± 1.0 | 17.6 ± 2.8 | 15.1 ± 1.8 | 1.03 ± 0.06 | 1.06 ± 0.06 | 1.66 ± 0.10 | 1.59 ± 0.08 |

F-CSRTT pre- and post-operative baseline data for animals with PFC and NAcSh noradrenergic lesions. Data expressed as the mean of three consecutive days prior to surgery (pre-), and prior to the first experimental manipulation (post-). Results are shown for the total population, mean ± SEM, PFC n=16 and NAcSh n=18, animals per group.
