# Supplementary material for: Differential roles for cortical versus sub-cortical noradrenaline and modulation of impulsivity in the rat
Source: Psychopharmacology (Berl). 2016 Oct 15;234(2):255–66. doi: 10.1007/s00213-016-4458-8 (PMC5203835; doi:10.1007/s00213-016-4458-8)
Supplement: Supplementary file 2 — (DOC 79 kb) [file 213_2016_4458_MOESM2_ESM.doc]

**Table S2: Regional assessment of DβH immunostaining**

|  |  | Area of DβH staining (mm^2^) | | | |
| --- | --- | --- | --- | --- | --- |
| Brain Region | Stereotaxic Level | PFC Sham | PFC Lesion | NAcSh Sham | NAcSh Lesion |
| Caudate putamen | 0.96 to 1.32 | 0.39 ± 0.06 | **0.16 ± 0.04*** | 0.27 ± 0.04 | 0.28 ± 0.04 |
| Cingulate cortex 1 | 1.08 to 3.72 | 32.56 ± 2.87 | **8.98 ± 1.73*** | 32.18 ± 1.31 | 31.30 ±1.53 |
| Cingulate cortex 2 | 1.08 to 1.32 | 17.82 ± 1.31 | **6.22 ± 1.24*** | 16.52 ± 0.85 | 14.60 ±1.53 |
| Dentate gyrus | -4.36 | 11.94 ± 1.26 | 10.80 ± 1.13 | 9.23 ± 0.76 | 7.26 ± 0.68 |
| Dorsal raphe nucleus, dorsal | -7.44 | 11.56 ± 1.32 | 16.35 ± 2.33 | 9.10 ± 1.26 | 10.86 ± 1.38 |
| Dorsal raphe nucleus, ventral | -7.44 | 6.04 ± 1.01 | 8.45 ± 0.80 | 5.13 ± 0.92 | 6.08 ± 0.50 |
| Hypothalamus, anterior | -1.72 | 9.90 ± 1.94 | 13.26 ± 1.67 | 9.12 ± 1.74 | 9.09 ± 3.21 |
| Infralimbic cortex | 2.76 to 3.72 | 18.56 ± 1.46 | **6.22 ± 1.16*** | 21.13 ± 1.29 | 21.61 ± 1.47 |
| Locus coeruleus | -9.60 to -10.08 | 194.07 ± 11.43 | 164.69 ± 10.92 | 210.95 ± 28.51 | 243.17 ± 9.66 |
| Lateral septal nucleus | 1.32 | 16.92 ± 1.25 | 16.81 ± 1.51 | 7.69 ± 1.07 | 6.84 ± 1.39 |
| Motor cortex 1 | 1.08 to 3.72 | 34.68 ± 3.32 | **11.62 ± 2.36*** | 34.72 ± 1.46 | 35.43 ±2.27 |
| Motor cortex 2 | 1.08 to 3.72 | 30.70 ± 3.17 | **10.51 ± 2.18*** | 29.74 ± 1.55 | 28.38 ± 1.24 |
| Median raphe nucleus | -7.44 | 5.40 ± 1.47 | 4.80 ± 0.69 | 5.50 ± 1.13 | 2.99 ± 0.61 |
| Nucleus accumbens core | 1.08 to 2.76 | 1.22 ± 0.21 | 1.37 ± 0.16 | 1.69 ± 0.20 | 1.35 ± 0.18 |
| Nucleus accumbens shell | 0.96 to 2.76 | 21.84 ± 2.61 | **11.67 ± 1.47*** | 21.16 ± 2.70 | **5.36 ±0.65*** |
| Orbital cortex, dorsolateral | 4.68 | 8.32 ± 0.74 | **4.03 ± 1.03*** | 8.38 ± 0.55 | 8.67 ± 0.74 |
| Orbital cortex, lateral | 3.72 | 9.20 ± 0.60 | **3.19 ± 0.51*** | 10.31 ± 0.79 | 8.75 ±0.94 |
| Orbital cortex, medial | 4.68 | 8.59 ± 0.27 | **5.48 ± 1.06*** | 9.05 ± 0.81 | 9.49 ± 1.25 |
| Orbital cortex, ventral | 3.72 to 4.68 | 17.58 ± 1.44 | **7.88 ± 1.19*** | 17.87 ± 0.73 | 15.00 ± 1.84 |
| Periaqueductal grey, p1 | -4.36 | 14.27 ± 3.47 | 17.98 ± 6.10 | 15.23 ± 1.83 | 19.28 ± 2.82 |
| Periaquaductal grey, dorsolateral | -7.44 | 6.82 ± 0.91 | 5.41 ± 0.70 | 4.72 ± 0.43 | 3.52 ± 0.46 |
| Periaqueductal grey, dorsomedial | -7.44 | 2.94 ± 0.56 | 2.75 ± 0.71 | 2.31 ± 0.29 | 1.38 ± 0.53 |
| Perirhinal cortex | -4.36 | 9.62 ± 1.13 | **5.47 ± 1.03*** | 9.60 ± 0.66 | 7.74 ± 0.69 |
| Prelimbic cortex | 2.76 to 4.68 | 22.30 ± 1.62 | **6.94 ± 1.74*** | 28.77 ± 1.06 | 27.51 ±2.02 |
| Thalamic nucleus, anteromedial | -1.72 | 11.80 ± 1.87 | 10.47 ± 2.45 | 17.62 ± 6.19 | 18.44 ± 1.76 |
| Thalamic nucleus, centralmedial | -1.72 | 6.89 ± 0.43 | 7.67 ± 1.90 | 5.94 ±1.33 | 5.06 ± 2.10 |
| Thalamic nucleus, paraventricular | -1.72 | 17.27 ± 3.28 | 16.34 ± 2.08 | 16.95 ± 2.94 | 18.70 ± 2.12 |

Area of DβH-immunoreactivity across different brain regions following PFC or NAcSh noradrenergic lesions. Averaged values were used for instances where multiple stereotaxic levels were analyzed, data presented as the mean ± SEM for each group, PFC (sham n=8, lesion n=8) and NAcSh (sham n=9, lesion n=9), *p<0.05, versus sham (within-subject). Stereotaxic levels according to bregma (Paxinos and Watson 2007).
