# Supplementary material for: Differential roles for cortical versus sub-cortical noradrenaline and modulation of impulsivity in the rat
Source: Psychopharmacology (Berl). 2016 Oct 15;234(2):255–66. doi: 10.1007/s00213-016-4458-8 (PMC5203835; doi:10.1007/s00213-016-4458-8)
Supplement: Supplementary file 3 — (DOC 44 kb) [file 213_2016_4458_MOESM3_ESM.doc]

**Table S3 : Statistical results from task manipulations**

| Group | | VITI | Noise |
| --- | --- | --- | --- |
| PFC | Premature | *t*_(10.5)_ = 1.47, *p* = 0.172 | *t*_(8.3)_ = 1.49, *p* = 0.159 |
|  | Correct | *t*_(14)_ = -0.84, *p* = 0.41 | *t*_(14)_ = -1.25, *p* = 0.233 |
|  | Omissions | *t*_(14)_ = 0.84, *p* = 0.41 | *t*_(14)_ = 1.25, *p* = 0.233 |
|  | Correct Latency | *t*_(14)_ = -1.10, *p* = 0.292 | *t*_(14)_ = -1.45, *p* = 0.171 |
|  | Collection Latency | *t*_(14)_ = 0.82, *p* = 0.425 | *t*_(14)_ = 0.71, *p* = 0.488 |
| NAcSh | Premature | *t*_(16)_ = 0.63, *p* = 0.537 | *t*_(16)_ = -1.25, *p* = 0.229 |
|  | Correct | *t*_(16)_ = -0.52, *p* = 0.613 | *t*_(16)_ = 0.29, *p* = 0.773 |
|  | Omissions | *t*_(16)_ = 0.52, *p* = 0.613 | *t*_(16)_ = -0.29, *p* = 0.773 |
|  | Correct Latency | *t*_(13.3)_ = 0.12, *p* = 0.910 | *t*_(16)_ = -0.48, *p* = 0.639 |
|  | Collection Latency | *t*_(16)_ = 0.92, *p* = 0.372 | *t*_(16)_ = 0.81, *p* = 0.431 |

Statistics reported for VITI and noise distractor manipulations in the F-CSRTT. Unpaired *t-*test for PFC (total n=16) and NAcSh (total n=18) groups, sham versus lesion.
