# Supplementary material for: Differential roles for cortical versus sub-cortical noradrenaline and modulation of impulsivity in the rat
Source: Psychopharmacology (Berl). 2016 Oct 15;234(2):255–66. doi: 10.1007/s00213-016-4458-8 (PMC5203835; doi:10.1007/s00213-016-4458-8)
Supplement: Supplementary file 4 — (DOC 63 kb) [file 213_2016_4458_MOESM4_ESM.doc]

**Table S4 : Statistical results from drug experiments.**

| Drug | Group | Variable | Group Effect | Dose*Group Interaction |
| --- | --- | --- | --- | --- |
| Atomoxetine | PFC | Premature | *F*_(1.0, 14.0)_ = 0.37, *p* = 0.553 | *F*_(3.0, 42.0)_ = 0.26, *p* = 0.851 |
|  |  | Correct | *F*_(1.0, 14.0)_ = 0.12, *p* = 0.736 | *F*_(2.2, 34.6)_ = 0.79, *p* = 0.454, ɛ = 0.74 |
|  |  | Omissions | *F*_(1.0, 14.0)_ = 0.12, *p* = 0.736 | *F*_(2.2, 34.6)_ = 0.79, *p* = 0.454, ɛ = 0.74 |
|  |  | Correct Latency | *F*_(1.0, 14.0)_ = 0.07, *p* = 0.800 | *F*_(3.0, 42.0)_ = 0.17, *p* = 0.918 |
|  |  | Collection Latency | *F*_(1.0, 14.0)_ = 1.12, *p* = 0.307 | *F*_(3.0, 42.0)_ = 0.33, *p* = 0.807 |
|  | NAcSh | Correct | *F*_(1.0, 16.0)_ = 1.74, *p* = 0.205 | *F*_(2.2, 34.6)_ = 0.89, *p* = 0.425 |
|  |  | Omissions | *F*_(1.0, 16.0)_ = 1.74, *p* = 0.205 | *F*_(2.2, 34.6)_ = 0.89, *p* = 0.425 |
|  |  | Correct Latency | *F*_(1.0, 16.0)_ = 2.98, *p* = 0.104 | *F*_(1.0, 16.0)_ = 2.98, *p* = 0.104 |
|  |  | Collection Latency | *F*_(1.0, 16.0)_ = 1.44, *p* = 0.248 | *F*_(1.0, 16.0)_ = 1.44, *p* = 0.248 |
| Amphetamine | PFC | Correct | *F*_(1.0, 13.0)_ = 0.15, *p* = 0.704 | *F*_(1.5, 19.4)_ = 0.95, *p* = 0.378, ɛ = 0.75 |
|  |  | Omissions | *F*_(1.0, 13.0)_ = 0.15, *p* = 0.704 | *F*_(1.5, 19.4)_ = 0.95, *p* = 0.378, ɛ = 0.75 |
|  |  | Correct Latency | *F*_(1.0, 13.0)_ = 0.06, *p* = 0.818 | *F*_(2.0, 26.0)_ = 0.95, *p* = 0.400 |
|  |  | Collection Latency | *F*_(1.0, 13.0)_ = 0.82, *p* = 0.383 | *F*_(1.3, 17.4)_ = 0.06, *p* = 0.877, ɛ = 0.67 |
|  | NAcSh | Premature | *F*_(1.0, 15.0)_ = 0.98, *p* = 0.337 | *F*_(1.6, 24.4)_ = 0.17, *p* = 0.806 |
|  |  | Correct | *F*_(1.0, 15.0)_ = 1.63, *p* = 0.221 | *F*_(1.3, 19.0)_ = 1.68, *p* = 0.214 |
|  |  | Omissions | *F*_(1.0, 15.0)_ = 1.63, *p* = 0.221 | *F*_(1.3, 19.0)_ = 1.68, *p* = 0.214 |
|  |  | Correct Latency | *F*_(1.0, 15.0)_ = 0.37, *p* = 0.552 | *F*_(1.6, 24.7)_ = 3.23, *p* = 0.18 |
|  |  | Collection Latency | *F*_(1.0, 15.0)_ = 0.36, *p* = 0.558 | *F*_(2, 30)_ = 0.58, *p* = 0.568 |

Statistics reported for atomoxetine and amphetamine dose response experiments. Repeated measures ANOVA with GROUP as the between-subject factor, and DOSE as a within-subject factor. Total (sham and lesion) number of animals per group; atomoxetine PFC n=16 and NAcSh n=18, amphetamine PFC n=15 and NAcSh n=17. Epsilon values have been displayed in instances where the degrees of freedom have been altered to more conservative values for sphericity violation according to Mauchly’s test.
